# Mitochondrial Deoxyribonucleic Acid (mtDNA), Maternal Inheritance, and Their Role in the Development of Cancers: A Scoping Review

**DOI:** 10.7759/cureus.39812

**Published:** 2023-06-01

**Authors:** Sabitha Vadakedath, Venkataramana Kandi, Jayashankar CA, Swapna Vijayan, Kushal C Achyut, Shivani Uppuluri, Praveen Kumar K Reddy, Monish Ramesh, P Pavan Kumar

**Affiliations:** 1 Biochemistry, Prathima Institute of Medical Sciences, Karimnagar, IND; 2 Clinical Microbiology, Prathima Institute of Medical Sciences, Karimnagar, IND; 3 Internal Medicine, Vydehi Institute of Medical Sciences and Research Centre, Bengaluru, IND; 4 Pediatrics, Sir CV Raman General Hospital, Bengaluru, IND; 5 Internal Medicine, Vydehi Institute of Medical Sciences and Research Centre, Bangalore, IND; 6 General Medicine, Vydehi Institute of Medical Sciences and Research Centre, Bengaluru, IND

**Keywords:** cancer, inheritance, mtdna, endosymbiotic theory, mitochondrial dna

## Abstract

Mitochondrial DNA (mtDNA) is a small, circular, double-stranded DNA inherited from the mother during fertilization. Evolutionary evidence supported by the endosymbiotic theory identifies mitochondria as an organelle that could have descended from prokaryotes. This may be the reason for the independent function and inheritance pattern shown by mtDNA. The unstable nature of mtDNA due to the lack of protective histones, and effective repair systems make it more vulnerable to mutations. The mtDNA and its mutations could be maternally inherited thereby predisposing the offspring to various cancers like breast and ovarian cancers among others. Although mitochondria are considered heteroplasmic wherein variations among the multiple mtDNA genomes are noticed, mothers can have mitochondrial populations that are homoplasmic for a given mitochondrial mutation. Homoplasmic mitochondrial mutations may be transmitted to all maternal offspring. However, due to the complex interplay between the mitochondrial and nuclear genomes, it is often difficult to predict disease outcomes, even with homoplasmic mitochondrial populations. Heteroplasmic mtDNA mutations can be maternally inherited, but the proportion of mutated alleles differs markedly between offspring within one generation. This led to the genetic bottleneck hypothesis, explaining the rapid changes in allele frequency witnessed during the transmission of mtDNA from one generation to the next. Although a physical reduction in mtDNA has been demonstrated in several species, a comprehensive understanding of the molecular mechanisms is yet to be demonstrated. Despite initially thought to be limited to the germline, there is evidence that blockages exist in different cell types during development, perhaps explaining why different tissues in the same organism contain different levels of mutated mtDNA. In this review, we comprehensively discuss the potential mechanisms through which mtDNA undergoes mutations and the maternal mode of transmission that contributes to the development of tumors, especially breast and ovarian cancers.

## Introduction and background

Mitochondrial deoxyribonucleic acid (mtDNA) is a small circular DNA found within mitochondria present in the cytoplasm of a cell. This DNA is supplementary to the nucleic acid material found in the nucleus of each cell. The mtDNA codes for 37 genes that promote the proper functioning of some cells. The mitochondria synthesize adenosine triphosphate (ATP) through oxidative phosphorylation and encode information for the synthesis of enzymes, transfer ribonucleic acid (tRNA), and ribosomal RNA (rRNA) [1]. Disorders of mtDNA and mutations in its genes can predispose to health problems like age-related hearing loss, diabetes, and brain, heart, and liver failure, among other conditions [2]. Moreover, mtDNA and its associated mitochondrial disorders can predispose people to different types of cancers including lymphomas, leukemias, and breast, intestine, liver, and kidney tumors, among others [1, 3]. However, the mechanisms behind carcinogenesis are not yet adequately elaborated. 

**Figure 1:**
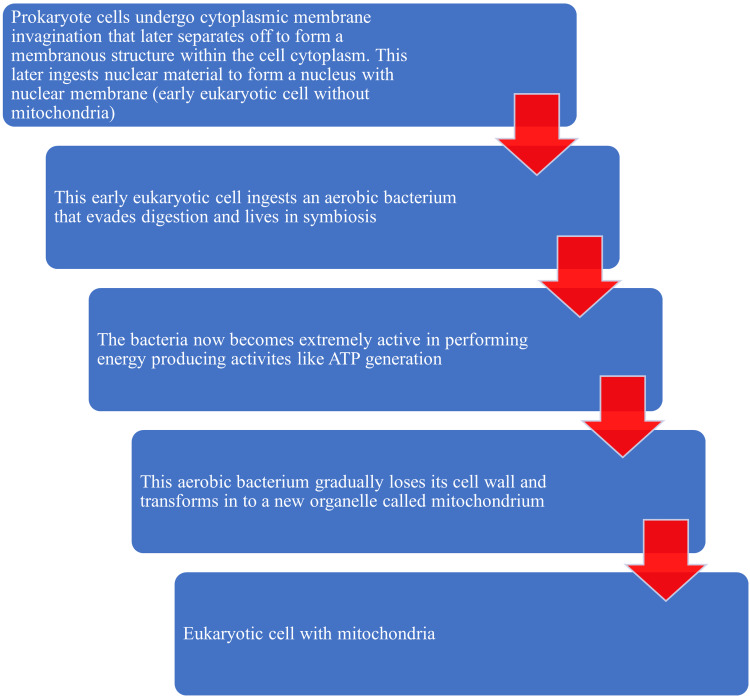
Endosymbiont theory depicting the evolution of mitochondria from prokaryotes. Note: This figure has been created by the authors ATP, adenosine triphosphate

From the results of evolutionary and genetic studies, mtDNA has been shown to be inherited from the mother during fertilization [4]. Additionally, studies have observed some important characteristics of mtDNA like high mutation rates, increased copy numbers in a cell, and unable to undergo recombination [5-7]. According to endosymbiotic theory, the origin of eukaryotic cells has been tracked back to the prokaryotes. It was Lynn Margulis who proposed the endosymbiotic theory in the 1960s [8]. This theory puts forth a gradual process that occurs over a long period of time for the development of eukaryotic cells from the prokaryotes [9]. The mitochondria, plastids, and chloroplasts could have descended from free-living prokaryotes [9]. The endosymbiotic theory presumes that complex multicellular organisms including humans have a small mitochondrial genome that is being generated and transferred through evolution, probably from the prokaryotes [10]. It has been hypothesized that some of the organelles of eukaryotic cells could have developed from the prokaryotes like bacteria. The early eukaryotic cells lacked mitochondria, but during evolution, these cells may have ingested the aerobic bacteria that had started living in symbiosis. During the course of evolution, the ingested aerobic bacteria lost their cell walls and may have developed into mitochondria [11]. The diagrammatic representation of the proposed evolution of mtDNA is shown in Figure 1.

## Review

Human mtDNA contains 16,569 base pairs and encodes 13 proteins. The mtDNA has a heavy strand and a light strand wherein the heavy strand is guanine rich and encodes 12 subunits. The light strand is rich in cytosine and encodes one subunit that performs oxidative phosphorylation. The regulation of the entire mtDNA genome is at the site of origin of replication of these strands [12]. The hyper diversity is the reason for the high mutational rate seen in mtDNA and vice versa. This hyper-diverse mtDNA causes unusual variations in DNA sequence, promoting coding variations, gene rearrangements, recombination, and finally high mtDNA substitution rates [13]. The initial transcription of mtDNA takes place on a displacement loop (D-loop) that has unusual bases like dihydro uracil, and hence it is the more susceptible region to mutations [14-15].

Generally, the hypervariable regions help in the anchoring of proteins to the membrane, protein-protein interactions, and protein signaling not specific to mtDNA. These regions have variations in tandem repeats seen mostly on the D-loop of mtDNA. Tandem repeats are frequently observed sequences on DNA. They are more prone to mispairing of bases known as ‘slipped strand mispairing.’ This may be the reason for the inappropriate mismatch repair (MMR) seen in mtDNA [16]. It was assumed that the eukaryotic mtDNA has an inefficient repair system but recent identification of the mutHLS (consisting of mutH, mutL, and mutS mutation detection and repair proteins) system has confirmed the potential role of mutHLS system in the repair process. Within the mtDNA, mutHLS repairs mismatch, strand break, insertion, and deletion of bases repair through endonuclease activity [17-18]. 

**Figure 2:**
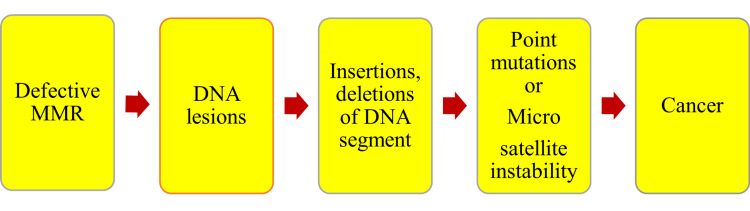
Effects of mismatch repair on mitochondrial DNA mutations and cancer. Note: This figure has been created by the authors MMR, mismatch repair; DNA, deoxyribonucleic acid

Less efficient MMR is seen in mtDNA compared to nuclear DNA [19]. Therefore, mtDNA could suffer from lesions, undergo deletions and insertions, and develop mutations that could predispose to carcinogenesis. The identification of mismatches and initiation of repair is very much required for genome stability. Defective MMR leads to the loss of gene activities. This further leads to microsatellite instability, elevates spontaneous mutation rate, and accelerates tumor development and progression as shown in Figure 2.

**Figure 3:**
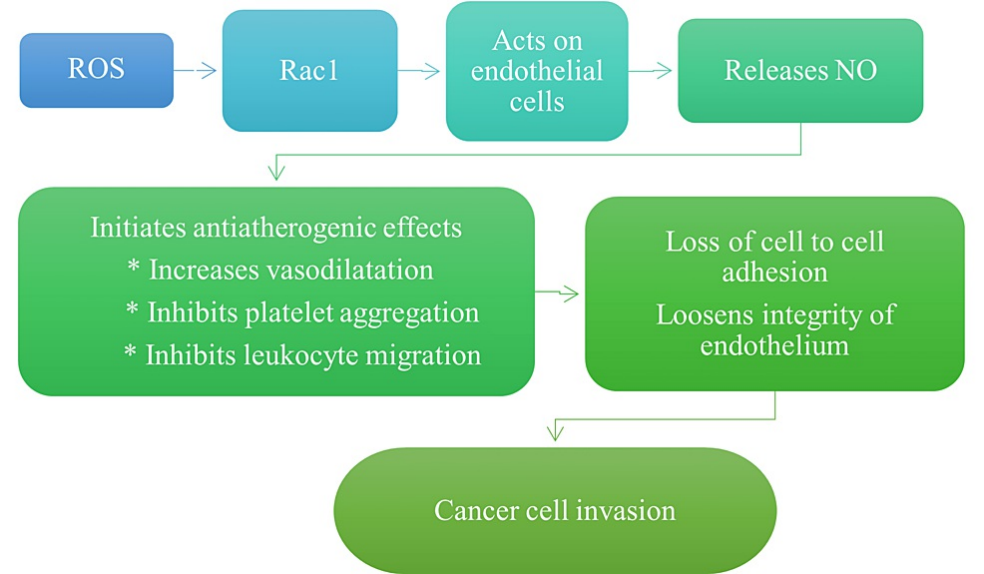
Role of ROS in the development of cancer. Note: This figure has been created by the authors ROS, reactive oxygen species; Rac1, Ras-related C3 botulinum toxin substrate 1; NO, nitric oxide

Another mechanism by which the defects in mtDNA can cause carcinogenesis is the generation of reactive oxygen species (ROS) during the process of oxidative phosphorylation [20-21]. Production of ROS by the mitochondria further leads to alterations in cellular vitality and metabolite concentrations. In this process, the ROS stimulates the Rac1 (Ras-related C3 botulinum toxin substrate 1) protein which acts on the vascular endothelial cells. This causes the release of nitric oxide (NO) that results in antiatherogenic effects like vasodilatation and inhibition of platelet and leukocyte aggregation. Moreover, this process results in the loss of cell-to-cell adhesion and loosens the endothelial integrity, and transforms the normal cells into cancer cells. The method of ROS-dependent carcinogenesis is shown in Figure 3.

The nuclear genome supplies protein for mitochondrial replication and gene expression. The mutations to these nuclear genomes not only alter mitochondrial function but also influences carcinogenesis and progression. It was found that the D-loop region and mitochondrial cytochrome b (MT-CYB) gene are specific sites of mtDNA where mutations are common [22-23]. The D-loop of mtDNA is required for transcription and gene expression. The strand/primer for initiation of transcription provides an additional strand that makes it a triple-strand DNA structure. This makes mtDNA more susceptible to the mutation of somatic cells that invariably lead to cancers [24]. This influences copy number of cells (variations in a repeated sequence of genes) as well as the regulation of mitochondria.

**Figure 4:**
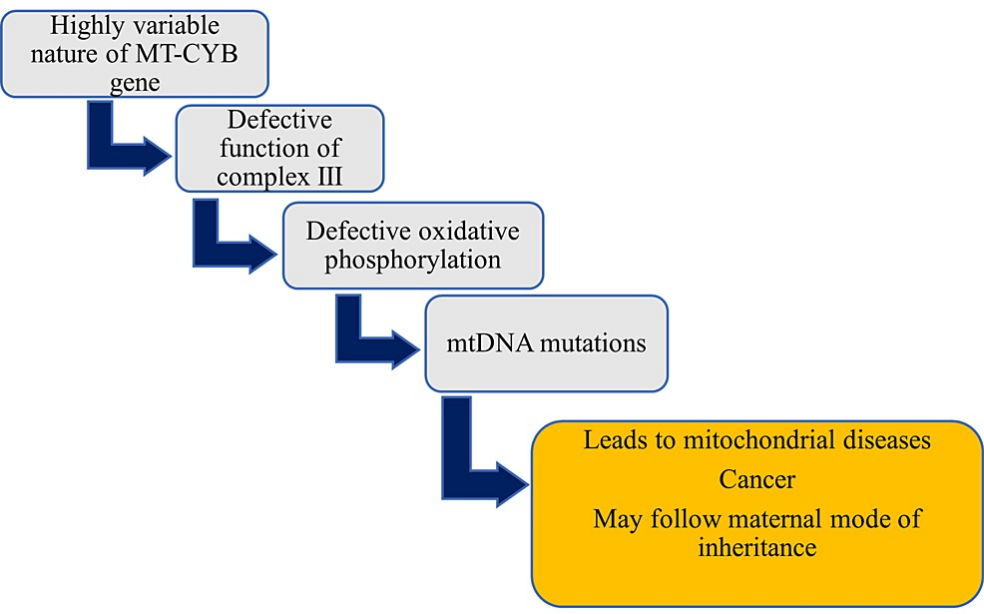
Role of MT-CYB gene in the development of mitochondrial diseases and cancer. Note: This figure has been created by the authors MT-CYB, mitochondrial cytochrome b; mtDNA, mitochondrial DNA

The MT-CYB gene provides the necessary information for the synthesis of cytochrome b protein of complex III involved in oxidative phosphorylation [20, 23]. This highly variable nature of the MT-CYB gene is also the reason for the development of cancers. It was identified that oral cancers are primarily due to mtDNA mutations [23]. But it is unclear whether it is D-loop region or MT-CYB gene mutations that lead to pathological consequences in cancer. Hence it was suggested that mtDNA could be used as a biomarker for the early identification of cancers [23]. The role of the MT-CYB gene in the development of mitochondrial diseases and cancers is shown in Figure 4.

mtDNA and maternal inheritance

The mitochondrial genome is independent of its inheritance pattern compared to chromosomal inheritance. Recent research had identified that paternal mtDNA is eliminated through various mechanisms which include mitophagy, and autophagy-independent degradation, among others. In mitophagy, aged and dysfunctional mitochondria are removed by lysosomal activity and/or proteasomal pathways at the embryonal stage [25-26]. Animal studies have suggested that the paternal mitochondria and the mtDNA could be eliminated by autophagosome-based sperm organelle mechanisms [27-29]. Other mechanisms like mitochondrial endonuclease-mediated elimination of paternal mtDNA have also been implicated in the maternal inheritance of mtDNA [30].

This enables uniparental inheritance which in turn keeps deleterious mutations of DNA genes at their location whilst restricting their spread [31-32]. Mitochondrial DNA (mtDNA) also contributes to the inheritance of cancer-susceptible alleles, loss of heterozygosity (two different genes) of inherited and mutated susceptible genes, and inactivation of tumor suppressor genes by deletion, insertion, or gene rearrangement. Additionally, biallelic inactivation (recessive), and heteroplasmy of mtDNA hold both abnormal and normal mitochondrial genomes due to unequal segregation [33-34]. The heteroplasmic nature of mtDNA leads to a genetic bottleneck which predicts that little mtDNA is transferred to the next generation limiting genetic variations in the family [35-36].

In a recent observation, it has been identified that the mitochondria meet the endoplasmic reticulum (ER) for the exchange of nutrients like calcium and glucose. Additionally, ER has been noted to assist in mitochondrial fusion and fission. Dysplasia of mitochondria in the neurons was found to predispose children to hereditary conditions like spastic paraplegias [37-38].

Influence of mtDNA in ovarian cancer and breast cancer

Current evidence suggests that there is an increasing frequency of hereditary breast and ovarian cancers [39]. Additionally, there is an increased awareness of gene mutations that could predispose to the familial transfer of cancers [40]. However, there is a scarcity of such information regarding the role of mtDNA and its influence on the development of cancers. The D-loop of mtDNA is more prone to mutations in breast and ovarian cancers. The mitochondrial microsatellite instability (mtMSI) and variations in CA (cytosine, adenine) repeats were also observed in ovarian and breast carcinoma. The inefficient MMR after malignant transformation may contribute to mtMSI. The mtMSI is observed in mononucleotide repeats at nucleotide positions 303-309. This region is the site where replication primer binds, making a defective DNA repair system. In a previous study, it was found that homo polymorphic nucleotide (variant forms of specific nucleotide sequence) tracts of mtDNA are more prone to errors due to the low frameshift (addition/deletion of bases) mutations. Moreover, the fidelity of DNA polymerase gamma (PLOG) that carries out the mtDNA replication process is lower than that found in the nucleus and is not severely affected [41-46].

In a recent report, it was noticed that mitochondria may play a significant role in the development, metastasis, chemotherapy resistance, and treatment of ovarian cancers [47]. The mitochondrial ribosomal proteins (MRPs) including the MRPL15 were found to be associated with the development of ovarian cancer. Moreover, this protein was found to be efficient in predicting the prognosis [48]. Damage to the mitochondria present in the ovarian germ and somatic cells may lead to the transfer of mitochondrial non-coding RNAs (ncRNAs) into the nucleus. This could predispose to early ovarian aging and resultant adverse effects including hormonal imbalances and tumorigenesis [49].

The abnormal transmission of mtDNA into the nuclear DNA was found to correlate with the development of breast cancer [50]. An association of mitochondrial D310 mutation was found to correlate with the development of various solid organ cancers including breast cancer [51].

Current research perspectives

Two different mtDNA variants/mutations were identified wherein the mutations that cause the formation of tumors are called inducers and those mtDNA mutations that facilitate the survival of tumor cells are called adaptors. The mtDNA mutations may be of three types based on their origins including those which are inherited and undergo familial transfer, mutations that happen within the cell, and ancient but transferable mtDNA variations [52]. Recent research had also suggested that better mitochondrial health can be instrumental in preventing replication errors and regulating apoptotic activities [53].

Studies have noted that mtDNA mutations, dysregulation of mtDNA, and disturbances in the communication between nuclear DNA and mtDNA may affect cellular health and in turn, predispose to carcinogenesis [54]. A recent hypothesis also suggested that the mitochondria may under the influence of microbial pathogens can transform themselves into super-power cells or immortal cancer cells that enable tumor formation [55]. In view of the potential role of mtDNA in aging and diseases including cancers, the role of gene editing in therapeutic management was being explored [56].

The aldehyde dehydrogenase 2 (ALDH2), a mitochondrial enzyme was found to regulate acetaldehyde toxicity within the cell and was suggested as a potential biomarker for diagnosis, metastasis, and prognosis of cancer. Additionally, abnormalities in ALDH2 and associated gene polymorphisms (single nucleotide polymorphisms-rs671) were noted to influence tumorigenesis and therefore should be considered as potential therapeutic targets [57].

Drugs like nilotinib, salinomycin, tigecycline, and eupatilin among others targeting mitochondrial dysfunctional pathways are being explored for their role in the treatment and management of ovarian cancer [58]. Given that the functional characteristics of somatic mtDNA are not sufficiently elucidated, it is impractical to understand the variations and mutations of mtDNA and its relationship with cancers [59-61].

## Conclusions

The mtDNA is inherited uniparentally probably owing to the loss of paternal mtDNA during fertilization. The unique nature of mtDNA to synthesize its proteins and its independent inheritance patterns makes it a strong maternal factor. The unstable nature of replication machinery with a defective repair system makes mtDNA more prone to mutations and tumorigenesis. The inheritance patterns seen in ovarian and breast cancers are solely dependent on the frequency of mtDNA mutations and their ability to utilize different repair mechanisms in various tumor cells. Therefore, it is extremely essential for the researchers and the physicians involved in cancer diagnosis, treatment, and management to improve the understanding of the potential role of mtDNA in the development, diagnosis, prevention, and cure of cancers.
